# CAnceR IN PreGnancy (CARING) – a retrospective study of cancer diagnosed during pregnancy in the United Kingdom

**DOI:** 10.1038/s41416-024-02605-x

**Published:** 2024-02-21

**Authors:** M. A. Baxter, M. Denholm, S. J. Kingdon, S. Kathirgamakarthigeyan, S. Parikh, R. Shakir, R. Johnson, H. Martin, M. Walton, W. Yao, A. Swan, C. Samuelson, X. Ren, A. Cooper, H-L Gray, S. Clifton, J. Ball, G. Gullick, M. Anderson, L. Dodd, H. Hayhurst, M. Salama, R. Shotton, F. Britton, T. Christodoulou, A. Abdul-Hamid, A. Eichholz, R. M. Evans, P. Wallroth, F. Gibson, K. Poole, M. Rowe, J. Harris, National Oncology Trainees Collaborative for Healthcare Research (NOTCH)

**Affiliations:** 1 Division of Molecular and Clinical Medicine, Ninewells Hospital and Medical School, University of Dundee, Dundee, UK; 2 Tayside Cancer Centre, Ninewells Hospital and Medical School, NHS Tayside, Dundee, UK; 3 Department of Oncology, Cambridge University Hospitals NHS Foundation Trust, Cambridge, UK; 4 Early Cancer Institute, Department of Oncology, University of Cambridge, Cambridge, UK; 5 Exeter Oncology Centre, Royal Devon University Hospitals NHS Trust, Exeter, UK; 6 Oncology Department, Nottingham University Hospitals NHS Trust, Nottingham, UK; 7 Department of Oncology, University Hospitals Birmingham NHS Foundation Trust, Birmingham, UK; 8 Oncology Department, Oxford University Hospitals NHS Foundation Trust, Oxford, UK; 9 Cancer Research UK Cambridge Institute, Cambridge University, Cambridge, UK; 10 Edinburgh Cancer Centre, Western General Hospital, NHS Lothian, Edinburgh, UK; 11 Bristol Haematology and Oncology Centre, Bristol, UK; 12 Oncology Department, Royal United Hospitals NHS Foundation Trust, Bath, UK; 13 Northern Centre for Cancer Care, The Newcastle Upon Tyne Hospitals NHS Foundation Trust, The Newcastle Upon Tyne, UK; 14 Department of Medical Oncology, The Christie NHS Foundation Trust, Manchester, UK; 15 Department of Oncology, Royal Surrey County Hospital NHS Trust, Surrey, UK; 16 Department of Oncology, Buckinghamshire Healthcare NHS Trust, Buckinghamshire, UK; 17 South West Wales Cancer Centre, Swansea Bay NHS Trust, Swansea, UK; 18 Mummy’s Star, Glossop, UK; 19 School of Health Sciences, Faculty of Health and Medical Sciences, University of Surrey, Guildford, UK; 20 Centre for Outcomes and Experience Research in Children’s Health, Illness and Disability, Great Ormond Street Hospital for Children NHS Foundation Trust, London, UK; 21 The Institute of Cancer Research, Clinical Trials and Statistics Unit, Belmont, Sutton, Surrey, UK; 22 Sunrise Oncology Centre, Royal Cornwall Hospitals NHS Trust, Truro, UK

**Keywords:** Cancer epidemiology, Epidemiology, Cancer epidemiology, Cancer epidemiology

## Abstract

**Background:**

The incidence of cancer diagnosed during pregnancy is increasing. Data relating to investigation and management, as well as maternal and foetal outcomes is lacking in a United Kingdom (UK) population.

**Methods:**

In this retrospective study we report data from 119 patients diagnosed with cancer during pregnancy from 14 cancer centres in the UK across a five-year period (2016-2020).

**Results:**

Median age at diagnosis was 33 years, with breast, skin and haematological the most common primary sites. The majority of cases were new diagnoses (109 patients, 91.6%). Most patients were treated with radical intent (96 patients, 80.7%), however, gastrointestinal cancers were associated with a high rate of palliative intent treatment (63.6%). Intervention was commenced during pregnancy in 68 (57.1%) patients; 44 (37%) had surgery and 31 (26.1%) received chemotherapy. Live births occurred in 98 (81.7%) of the cases, with 54 (55.1%) of these delivered by caesarean section. Maternal mortality during the study period was 20.2%.

**Conclusions:**

This is the first pan-tumour report of diagnosis, management and outcomes of cancer diagnosed during pregnancy in the UK. Our findings demonstrate proof of concept that data collection is feasible and highlight the need for further research in this cohort of patients.

## Introduction

Increasing numbers of women are being diagnosed with cancer during pregnancy [1–4]. This has been associated with the sustained global trend to delay conception in developed nations, including in the United Kingdom (UK), and the fact that the incidence of most malignancies increases with age [5, 6].

Estimates of cancer incidence during pregnancy are challenging as not all pregnancies result in live births and health registries do not routinely combine oncological and obstetric data. Despite this, a range of incidence rates between 17 per 100,000 live births and 25−27 per 100,000 pregnancies have been estimated [3]; equating to approximately two new cases per day in the UK.

Increased use of non-invasive prenatal testing as a screening test to detect foetal chromosomal abnormality, using cell free DNA from maternal blood, has been documented to have led to asymptomatic women being diagnosed with cancer during pregnancy [7]. Pregnancy may complicate diagnostic and therapeutic oncological options due to the need to consider the safety/implications for the unborn child. Pregnancy also presents considerable challenges around the decision-making required of parents and physicians regarding treatment and care options (both cancer and maternity-related) [8–10].

Usual tumour treatment protocols require adaptation by the cancer multidisciplinary team in the context of pregnancy and where possible should involve obstetric team and family doctor, as well as consider the women’s views and preferences. In terms of possible treatment modalities, UK guidance is provided by the Royal College of Obstetricians and Gynaecologists. In general terms, where appropriate, surgery can usually be undertaken at any trimester but may not be feasible depending on tumour location. Whilst radiotherapy is usually contraindicated it may be undertaken in specific circumstances (for example with the aim of preservation of life or function) with foetal shielding. Systemic chemotherapy should be avoided in the first trimester but is considered safe from the second trimester although birth should not be more than 2−3 weeks after the last chemotherapy to allow bone marrow recovery and reduce risk of neutropenia. Immunotherapies, hormone and targeted therapies are contraindicated until after delivery. After live birth, women should not breastfeed if being treated with chemotherapies, immunotherapies, hormone and targeted therapies, and may need to avoid their young infants and existing children if being treated with radiotherapy.

Due to the inherently threatening nature and complex challenges of being diagnosed with cancer during pregnancy, women and their families may require enhanced psychosocial and supportive care.

Internationally, research is gaining momentum in the field of cancer in pregnancy [3]; however, there is a lack of UK data. The primary source of UK data is limited to an analysis by Public Health England linking Cancer Registry and Hospital Episode Statistics [11]. There is currently no routine clinical data collection in the UK. As such, there is limited data about routes to and the timing of cancer diagnosis in relation to gestation or delivery. There is also minimal data relating to patient demographics, treatment decisions, healthcare interventions (both cancer and maternity) or outcomes for mothers and neonates.

To address this research gap, this project aimed to provide the UK’s first comprehensive assessment of cancer during pregnancy in women aged 16 years and older, including their oncological care and clinical outcomes. The objectives of the study were to:

1. Establish an estimate of the number of cases of cancer diagnosed during pregnancy in the UK over a five-year period including by the type of tumour, stage, recurrence rates and timing of diagnosis.
2. Describe the demographic and maternal profile of women diagnosed with cancer during pregnancy in the UK.
3. Describe treatments administered including by tumour type and by gestational age.
4. Describe maternal/infant outcomes in the immediate postnatal time (e.g., including maternal death and pregnancy outcomes. Long-term infant follow-up was not included).

We present the results in this manuscript, which we feel have huge potential to inform future research directions and clinical practice in the UK.

## Methods

### Study design

This was a retrospective study over a five-year period. Patients with cancer diagnosed during pregnancy between 1st January 2016 and 31st December 2020 were eligible for inclusion. The National Oncology Trainee Collaborative for Healthcare Research (NOTCH) network [12] was utilised for data collection at individual sites in England, Scotland and Wales. The data fields collected were mapped to the European International Network on Cancer Infertility and Pregnancy (INCIP) database [4] and selected according to the feasibility of collection within the UK National Health Service (NHS) Oncology and Obstetrics frameworks and patient pathway. The data capture template is available on request.

Data collection was focused on the nature of clinical presentation of cancer during pregnancy as well as subsequent oncological management and both maternal and foetal outcomes immediately post-partum (Supplementary Table 1). Patients were identified on a local level using available methods, which varied according to site; most commonly either clinician recall or use of electronic coding.

### Statistical analysis

Data from each site was collated by the primary investigator (MAB). Anonymised data were cleaned, derived and composite variables were computed (Supplementary Table 1) and analysed by an independent analyst (JH). Descriptive statistics are presented overall and by tumour type. To maintain patient anonymity, demographic categories (e.g., rarer tumour histology) with fewer than five patients, were grouped together prior to presentation of results. All analyses were performed using R Studio *version 4.3.2*. Code is available on request.

### Ethics

Local Caldicott Guardian approval was obtained at each individual site. As this was a retrospective study, patient consent was not required.

## Results

**Fig. 1:**
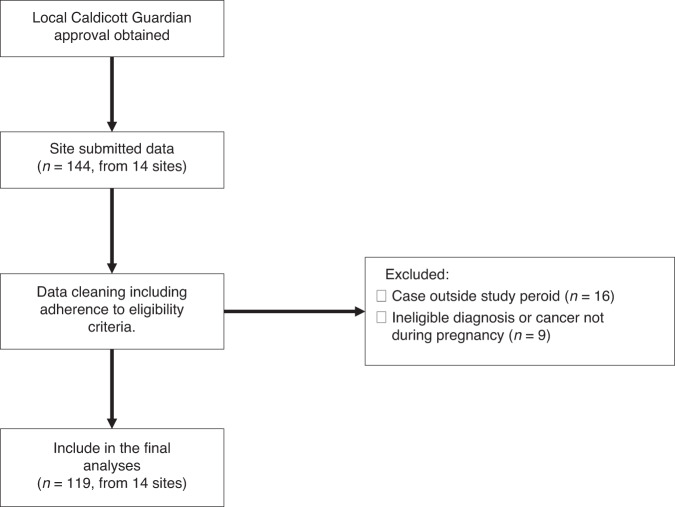
Study flow diagram and STROBE diagram of patient inclusion. *n* = number of patients.

Data were collected for 144 patients from 14 sites (NHS Trusts listed in Supplementary Table 2) during the study period. These sites included cancer treatment centres in England (10 sites), Scotland (3 sites) and Wales (1 site), serving an estimated population of 21 million. Of these 119 patients were eligible for analysis (Fig. 1). Data for 88 patients were collected from the five centres using electronic records. There was an even distribution of cases according to year of diagnosis (Supplementary Fig. 1) with a mean of 24 cases per year across all sites.

### Demographics

The demographics of the included patients are shown in Table 1. The median age at diagnosis was 33 years old (range 22−49). The most common primary sites were breast (25.2%) and haematological (15.1%). The majority of patients were symptomatic at diagnosis (82.4%) and the diagnosis was of a new malignancy (90%). Over half of women with genitourinary cancers were asymptomatic at diagnosis (51.1%), whereas symptomatic presentation was more common for other primary sites (range 80% for skin to 100% for gastrointestinal).

**Table 1:** Demographics of included population.

	*N*	%	
Demographic characteristics			
*Ethnic background*			
Any white background	91		76.5
Non-white background	13		10.9
Missing	15		12.6
Mean (SD); *median (IQR)* age^a^	33.6 (4.72)		*33* (30−37)
Clinical characteristics			
*Cancer Stage*^b^			
I	34	33.0	
II	18	17.5	
III	9	8.7	
IV	20	19.4	
NA	22	21.4	
*Cancer onset during pregnancy*			
New cancer	109	91.6	
Recurrent cancer	10	8.4	
*Estimated trimester at cancer diagnosis*			
1st	16	13.5	
2nd	46	38.6	
3rd	37	31.1	
Unknown	20	16.8	
*Treatment intent*			
Unknown	3	2.5	
Palliative	20	16.8	
Radical	96	80.7	
*Cancer diagnosis group*			
Breast	30	25.2	
Skin	25	20.0	
Haematological	18	15.1	
Gastrointestinal^c^	11	9.2	
Genitourinary^d^	14	11.8	
Other^e^	21	17.7	
*Initial presentation*			
Asymptomatic	21	17.7	
Symptomatic	98	82.4	
*Smoking status*			
Current smoker	7	5.9	
Ex-smoker	18	15.1	
Never smoked	59	49.6	
Unknown	35	29.4	
Mean (SD); *median (IQR)* BMI	26.9 (6.17)	24.9 (23.0, 30.9)	
Mean (SD); *median (IQR)* gravidity^e^	2.1 (1.31)	2 (1, 3)	
Mean (SD); *median (IQR)* parity^f^	1.1 (1.12)	1 (0, 2)	
*Conception and fertility*			
Assisted conception	7	5.9	
Natural	99	83.2	
Unknown	13	10.9	

*IQR* interquartile range, *SD* standard deviation.

^a^Age range 22−49.

^b^Excludes 6 for whom staging data were unavailable.

^c^Gastrointestinal includes upper and lower tract.

^d^Include ovarian, cervical and urological.

^e^Includes categories with fewer than 10 women including ear-nose-throat, lung, thyroid, neurological, unknown primary.

^f^Gravidity range 0−8.

^g^Parity range 0−4.

Included patients were primarily caucasian (76.5%) and approximately half had never smoked (49.6%). The median body mass index (BMI) was 24.9 kg/m² and the median gravidity and parity were 2 (range 0−8) and 1 (range 0−4, 34.2% were nulliparous) respectively. Relating to the pregnancy, most cases were natural conception (83.2%).

### Staging investigations

The majority of patients (56.7%) received more than one staging investigation. A quarter (23.3%) of patients had no cancer staging investigations recorded during their pregnancy (Table 2); however, most of these patients were diagnosed with skin cancer (20/28 patients, 71.4%) (Table 3). Of these, 11 were basal cell carcinoma and 9 malignant melanoma.

**Table 2:** Investigations and treatments during pregnancy.

	*N*	%
*Investigation type*^a^		
Ultrasound	55	46.2
Mammography	19	16.0
X-ray	21	17.7
MRI/Diffusion-weighted MRI	60	50.4
CT	16	13.5
PET CT	-	-
Bone scan	11	9.2
Endoscopy	8	6.7
*Number of investigations*^*b*^		
0	28	23.5
1	24	20.2
2	30	25.2
3	25	21.1
4+	12	10.1
*Treatment types*^a^		
Surgery	44	37.0
Abdominal/cervical surgery	21	17.7
Chemotherapy	31	26.1
Anthracyclines	14	11.8
Alkylating agent (excluding platinums)	19	16.0
Antimetabolites	9	7.6
Taxanes	18	15.1
Platinum	8	6.7
Other	7	5.9
Missing	-	-
Radiotherapy	-	-
Other therapies^c^	10	8.4
*Number of treatments*		
0	51	42.9
1	54	45.4
2+	14	11.7
Timing of surgery^d^		
1st trimester	5	13.5
2nd trimester	17	46.0
3rd trimester	15	40.5
Timing of chemotherapy^e^		
2nd trimester	17	68.0
3rd trimester	8	32.0

^a^Includes when patients may have had more than one treatment.

^b^Include all those defined under treatment type.

^c^Includes targeted therapy, immunotherapy, hormone therapy and steroids.

^d^Excludes 5 with data unavailable.

^e^Excludes 6 with data unavailable.

**Table 3:** Characteristics of patients according to tumour group.

	Breast (*n* = 30)		Skin (*n* = 25)		Haematological (*n* = 18)		GI (*n* = 11)		Genitourinary (*n* = 14)		Other (*n* = 22)	
	*N*	%	*N*	%	*N*	%	*N*	%	*N*	%	*N*	%
Mean (SD); *median (IQR)* age	34.8 (4.30)	35 (31, 38)	32.6 (4.82)	34 (32, 38)	30.8 (4.87)	31 (28, 33)	34.4 (3.13)	34 (31, 38)	32.9 (4.25)	32.5 (29, 37)	33.1 (5.28)	32.0 (31, 34)
*Asymptomatic presentation*	2	6.7	5	20.0	2	11.1	-	-	8	57.1	4	19.1
*Recurrent cancer*	4	13.3	2	8.0	1	5.6	-	-	1	7.1	2	9.5
*Investigations*												
Ultrasound	26	86.7	2	8.0	11	61.1	6	54.5	5	37.7	5	23.8
Mammography	17	56.7	-	-	-	-	-	-	-	-	2	9.5
X-ray	6	20.0	-	-	5	27.8	1	9.1	3	21.4	6	28.6
MRI	11	36.7	2	8.0	9	50.0	4	36.4	12	85.7	14	63.7
Diffusion-weighted MRI	7	23.3	2	8.0	6	33.3	7	63.6	3	21.4	9	42.9
CT	5	16.7	-	-	5	27.8	1	9.1	-	-	5	23.8
Bone scan	3	10.0	-	-	2	11.1	6	54.6	-	-	-	-
Endoscopy	3	10.0	2	8.0	3	16.7	-	-	--	-	-	-
*No investigations during*	1	3.3	20	80.0	2	11.1	1	9.1	2	14.3	2	9.52
*Treatment intent*												
Palliative	3	10	2	8.3	-	-	7	63.6	1	7.1	7	31.3
Radical	27	90	22	91.7	16	100	4	36.4	13	92.9	14	66.7
*Treatments*												
Surgery	12	40.0	16	64.0	-	-	5	45.5	5	35.7	6	27.6
Chemotherapy	15	50.0	-	-	8	44.4	4	36.4	4	28.6	-	-
Other therapies	3	10.0	-	-	4	22.2	-	-	-	-	3	14.3
*Birth outcome*												
Full-term birth	10	52.6	1	5.0	8	53.3	4	66.7	6	75.0	6	42.9
Live birth	26	89.6	22	88.0	16	89.0	7	63.6	9	64.3	18	90.0
*Birth mode caesarean section*	11	42.3	9	40.9	10	58.8	6	75.0	9	90.0	9	52.9
Mortality rate	6	20.0	2	8.0	2	11.1	5	45.5	1	7.1	8	38.1

Gastrointestinal (GI) includes upper and lower tract. Genitourinary include ovarian, cervical and urological. Other includes categories with fewer than 10 women including ear-nose-throat, lung, thyroid, neurological, unknown primary.

The primary modes of investigation during the staging pathway included ultrasound (46.7%) or MRI/diffusion-weighted MRI (50.4%). Of note, 13.5% of patients underwent a CT scan and 9.2% had a bone scan. No patients had a PET CT scan.

### Treatments

Most patients were diagnosed at an early stage and had a plan for curative intent treatment (80.7%). The treatments administered are detailed in Table 2; 26.1% had chemotherapy during pregnancy, with two-thirds (68.0%) having treatment during their second trimester. No patients received chemotherapy during the first trimester.

Overall, a third (36.7%) of patients had some form of surgery with this typically taking place in the second or third trimester (46.0% and 40.5%, respectively). Nearly half of patients had no treatment for their cancer during their pregnancy (42.9%); with 56.4% diagnosed during the third trimester having no (active) treatment, compared with 35.9% and 2.2% diagnosed with cancer during trimesters two and one, respectively (data not shown, but available on request). As expected, the types of anti-cancer treatment varied according to tumour type (Table 3).

### Maternal and foetal outcomes

At the date of censoring in each individual site, 24 (20.0%) patients had died due to disease progression. Of those alive (*n* = 95), 75 (78.9%) were in remission, 12 (12.6%) were on ongoing treatment and 8 (8.4%) were lost to follow-up (Table 4). In those who had died, patients with a gastrointestinal primary had the highest mortality rate (45.5%, Table 3); likely because of later stage at diagnosis (for 63.6% treatment intent was palliative, Table 3).

**Table 4:** Summary of outcomes.

	*N*	%
*Birth outcome*		
Live birth	98	81.7
Miscarriage	7	5.9
Termination of pregnancy	10	8.4
Other outcomes^a^	4	3.4
*Delivery mode*^*b*^		
Caesarean section	52	53.1
Vaginal delivery	46	47.0
Pregnancy term at delivery^c^		
Preterm	35	29.4
Full term	*47*	39.5
Unknown	37	31.1
*Maternal outcome by follow-up*		
Remission	75	63.0
Disease persistent/recurrent	12	10.1
Died	24	20.2
Unknown/lost to follow-up	8	6.7

^a^Other birth outcomes include live birth followed by subsequent neonatal death, stillbirth and unknown. Excludes 2 with data unavailable.

^b^For live births only.

^c^Full term birth estimated for those with any live or still birth with delivery from 37 weeks.

Relating to foetal outcomes, there were 98 (81.7%) live births, 10 (8.3%) terminations and 8 (6.7%) miscarriages (Table 4). For those live births over half were delivered by caesarean section (52 (53.1%), data not shown but available on request). There was a higher rate of incidence of caesarean section for gastrointestinal and genitourinary primaries (75.0% and 90.0% respectively, Table 3). For live births, 42.5% (*n* = 34) were estimated to be pre-term (<37weeks gestation) with 79% (*n* = 27) of pre-term births being delivered by Caesarean-section.

## Discussion

Cancer diagnosed during pregnancy is increasing in incidence and provides decisional challenges for both patients and clinicians. Despite a growth in research and availability of data in Europe [3], analysis of clinical data related to patients in the UK is lacking [11]. This study aimed to provide the first comprehensive retrospective cohort study of the characteristics, management and outcomes of patients diagnosed with cancer during pregnancy in the UK.

This study reports 119 patients, diagnosed with cancer from 14 UK centres over a five-year period between 2016 and 2020. The median age of the cohort (33 years) is similar to that observed in prior studies including several case series in individual tumour groups and a report of 1170 cases from the INCIP group [13–16]. The ethnicity data is in keeping with UK census data and national audits of the characteristics of all women who gave birth [17], suggesting this dataset is broadly representative of the UK population. Compared with the INCIP group, fewer of the UK cohort were first-time mothers (44–56% and 34.2%, respectively), which is notable because previous research suggests parity can be important factor for patients where there are decisions around continuation with pregnancy and delaying treatment, and has implications for managing treatments alongside existing parenthood [18].

As expected, the most common sites of malignancy were breast (25.2%), skin (21%) and haematological (15.1%), due to their younger age of onset and higher incidence in the general population. This is mostly consistent with the published literature [16] although a National Cancer Registry Audit found cervical cancer to be the third most prevalent (15.2% of all cases between 2012 and 2015) [11]. Our study also captured the data relating to several other primary sites—highlighting the broad spectrum of malignancy that can affect pregnancy.

The majority of cases were new diagnoses, however approximately 8.4% were a cancer recurrence. Of the 10 observed recurrences, four were from a breast primary (three of which were oestrogen receptor positive). This data highlights the need for close surveillance during pregnancy, particularly in those with a previous history of malignancy.

Most women were symptomatic, but more than half of women with genitourinary cancer were diagnosed asymptomatically. It is possible that these women were diagnosed as part of routine maternal screening programmes and further research is needed to understand pregnant women’s pathways to cancer diagnosis and the barriers and enablers to timely presentation [19].

Despite the majority of cases being diagnosed at early, and thus curative stage (80.7%), there were high rates of advanced disease in gastrointestinal cancers despite all of these cases presenting symptomatically; 63.6% treated with palliative intent. This has been reported previously with Kocian et al. reporting a rate of 41.5% Stage 4 disease for pregnant women with gastrointestinal cancers [15]. The reasons may be that these cancers traditionally present at later stage due to their vague symptom profile, but also many of the symptoms of these malignancies could be erroneously attributed to pregnancy e.g., fatigue, abdominal pain or gastroesophageal reflux, a process described as diagnostic overshadowing in the context of cancer delay [20].

Only a small proportion of patients received no staging investigation following diagnosis. For those that did, the main modes of investigation were non-ionising; although 16 (13.5%) women underwent a CT and 11 (9.2%) a bone scan. Both of these imaging modalities are not generally recommended for use due to the risk of foetal radiation exposure [21]. Future detailed research is needed to explore to what extent ionising scans in pregnancy adhered to specific safety guidance and if the benefits are likely to have outweighed the risks [22–24].

In relation to treatment, the majority of women had anti-cancer treatment during pregnancy (57.1%)—with those who did not, often diagnosed later in their pregnancy. No patients received radiotherapy. Over a third (37.0%) had surgery, of which half of cases were abdominal/cervical (21 out of 44, 47.7%), which are comparable rates to INCIP findings; however, only a quarter of the cohort were treated with chemotherapy (26.0%) compared with 37% reported by INCIP [16]. This may suggest an underutilisation of chemotherapy in a UK context and warrants further investigation.

It is difficult to draw conclusions regarding the impact of treatment on maternal outcomes, however, 1 in 5 women within the cohort died. The MBRRACE maternal mortality report of women in the UK who died during or within 42 days of the end of pregnancy between 2019-2020 indicates that during pregnancy breast, ovary and cervix cancers were indirect causes of death (rate 0.19 per 100,000 maternities) [25]. In our study, proportionally, cancers of the gastrointestinal tract had the highest mortality rate. This may reflect innately more aggressive disease biology of these tumours and/or be a consequence of their late presentation. International comparisons of our data are challenging as prognostic studies have typically focused on specific tumour types such as breast cancer. However, notably even for women with breast cancer, the crude mortality rate in this study 20% compared to 14% found by the INCIP study [26]. Whether this is linked to the apparent undertreatment of pregnant women with cancer in the UK compared to other countries requires further investigation.

The caesarean section rates were higher than found in the general UK population (reported rates in non-cancer population between 28-35%) [27]. Generally, vaginal delivery is recommended for women diagnosed with cancer during pregnancy [28] unless in the case of routine obstetric indications or pelvic tumours. In this study gastrointestinal malignancies, alongside genitourinary cancers had the highest rates of caesarean section (75% and 90% respectively) adhering to these recommendations. Of note, these tumour groups also had the lowest rates of live birth (64% for both). In this study, almost a third of deliveries were preterm (29.4%) which compares to an estimated 7.6% of all births in the UK [29] and three-times the rate reported in international registries (10%) [16]. These findings support calls for further exploration in the UK context as international research also suggests that preterm births, rather than chemotherapy exposure are likely to have a greater impact on neonatal development [3, 16, 30].

While considering the results of this study, we must acknowledge that numbers are small and are likely to represent only a proportion of women diagnosed with cancer during pregnancy at participating sites (we estimate 14.8% of approximately 800 women who would be expected, based on UK wide estimates and the population covered by these centres), therefore it is difficult to draw any firm conclusions. We must also recognise that the method of patient identification varied across the UK. Most centres relied on clinical recall, which is open to bias; however, 74% of the sample were derived from sites that were able to link cancer and pregnancy using their electronic patient records. In addition, some patients do not see an Oncologist/Haematologist following diagnosis, particularly those diagnosed at early stage. In these cases, care is often coordinated by obstetrics and/or surgical teams. In contrast, some centres had electronic coding available and were able to link pregnancy to cancer or cancer treatment. These findings are important feasibility considerations for any future study in the UK.

This study supports the urgent need for a national approach to linking obstetric and cancer data. Pregnancy is a protected characteristic under the UK Equality Act 2010 and it is essential that we are able to audit treatments and the outcomes for these women and their families, and that we have standardised datasets which are at least as good as those collected internationally. It is also important to note that the NOTCH network is primarily oncologists who treat solid organs cancers – therefore, the representation of patients with haematological malignancies may be lower than expected. Furthermore, as a preliminary study NOTCH investigators were not able to access the children’s notes and no foetal outcomes beyond the immediate postnatal period were available, which again are incorporated into international datasets such as INCIP.

However, despite the above, our study has several strengths. Due to the study design the data points were readily available and very little data were missing. Although a few data points had somewhat higher rates of missing data, for example ethnicity, the completeness of this data were comparable those typically observed in maternity services [17] and better than rates typically reported in studies using routine cancer data [31]. In addition, the data were entered manually by oncologists following review of case notes—as such we can presume it is reliable. To our knowledge, this is the first pan-tumour data relating to the topic from the UK. In addition, the collation of data from three of the four nations in the UK, covering a population of over 20 million suggests this is a representative sample.

In summary, in this manuscript we provide data relating to management and outcomes of cancer diagnosed during pregnancy in the UK over a five-year period. This study demonstrates proof of concept that collecting this data is feasible within the UK health system, which is important as the UK currently lags behind the progress other developed nations have made in establishing national datasets.

This study has identified a group of people with a range of different cancer types but with unique needs. We hope this data can inform future research directions and clinical practice in the UK. Possible avenues to improve data collection and to further inform clinical practice is the establishment of a National Pregnancy in Cancer Audit similar to the National Pregnancy in Diabetes Audit [32], whereby the occurrence of cancer during pregnancy is routinely coded alongside maternal, oncological and foetal/infant care and outcomes. This is probably best achieved through the establishment of a national clinical interest group comprised of multidisciplinary clinicians and researchers including stakeholder representation, for example from professional organisations and societies, policy makers and NHS Digital.

Although there may be utility to initially focus efforts on tumours where co-occurrence of cancer and pregnancy are likely to be higher and where UK clinical guidance has been published (such as breast and gynaecological), longer-term it will also be important to consider outcomes for patients affected by less common cancers where survival may be worse.

The data collection tools of INCIP which enable standardised collection of maternal, oncological and foetal/infant outcomes, were adapted for this study and found to be feasible to implement within the UK context and could provide the foundations for such a database. Furthermore, it will be important to involve supporting charities as well as mother’s and families affected by cancer during pregnancy, in formulating the primary research questions and database specification to ensure the audit includes what matters most to those affected by cancer during pregnancy.

This approach could facilitate the development of a national framework to contribute annual data to international data, compare UK data against other nations and to work collaboratively to review treatment/outcomes and experiences of patients and their families.

### Supplementary information

Supplementary Material

## Data Availability

Data is available on request to the corresponding author.
