# Supplementary material for: CAnceR IN PreGnancy (CARING) – a retrospective study of cancer diagnosed during pregnancy in the United Kingdom
Source: Br J Cancer. 2024 Feb 21;130(8):1261–8. doi: 10.1038/s41416-024-02605-x (PMC11014900; doi:10.1038/s41416-024-02605-x)
Supplement: Supplementary file 1 — Supplementary Material [file 41416_2024_2605_MOESM1_ESM.docx]

**Supplementary Table 1**

Data codebook: data collected and analysis variable definitions

| **Derived or composite variable descriptor** | **Data collected** | **Analysis variable** |
| --- | --- | --- |
| Ethnic background | 17 ethnic groups and other (with description) | Collapsed into White/ non-white/missing |
| Cancer stage | Number staging, TNM staging and freetext notes entered - independently coded by oncology clinician | Stage I/ II/ III/IV/NA |
| Estimated trimester at diagnosis | Calculated based on date of diagnosis and due date | 1^st^ trimester (<12 weeks)/  2^nd^ trimester (13-27 weeks)/  3^rd^ trimester (28-40+ weeks)/  Unknown (either date missing) |
| Cancer diagnosis group | 15 tumour groups and other with freetext note entered. Details about relevant subtype collected. Any with fewer than 10 cases were grouped into larger categorical grouping by oncology clinician | Breast/ Skin/ Haematological/ gastro intestinal (GI, including upper GI, colorectal etc)/ Genitourinary (includes ovarian, cervical and urological/ Other (includes ear-nose-throat, lung, thyroid, neurological, unknown primary) |
| Live birth | Some groups had few cases so to protect confidentiality they were aggregated. | Live birth miscarriage/ termination/ other (includes stillbirth, live birth followed by neonate death, unknown) |
| Estimated pregnancy term at delivery | Calculated based on due date and end of pregnancy date for those who did not miscarry or have termination of pregnancy | Preterm (<37 weeks)  Full term (≥37 weeks)  Unknown (either date missing) |
| Maternal outcome | 9 categories including Ongoing primary treatment, Complete remission, Persistent disease: partial remission, Persistent disease: stable disease, Persistent disease: progressive disease, Persistent disease: not specified, Recurrent disease  Patient died, Lost for follow up. Some groups had few cases so to protect confidentiality they were aggregated. | Remission / Disease persistent or recurrent/ Died/ Unknown/lost to follow-up |
| Investigation type | Each investigation was coded for presence/absence. Date was checked to ensure it was during pregnancy. Patients could have more than one investigation. Total number calculated. | Yes/No  Total number of investigations grouped as 0, 1, 2, 3, 4+ |
| Treatment type | Each treatment was coded for presence/absence. Date was checked to ensure it was during pregnancy. Patients could have more than one treatment. Total number calculated. | Yes/No  Total number of treatments grouped as 0, 1, 2+ |
| Surgery abdominal/cervical | For those with surgery, freetext notes about type of surgery checked and new variable created | Yes/No |
| Chemotherapy drugs | For those with chemotherapy, freetext notes about type of chemotherapy checked and new variable coded by oncology clinician in line with INCIP groupings | Anthracyclines/ Alkylating agent (excluding platinum)/ Antimetabolites/Taxanes/ Platinum/Other/ Missing |
| Timing of surgery | Calculated based on due date and timing of surgery, verified against diagnosis date and end of pregnancy date. |  |
| Timing of chemotherapy | Calculated based on due date and timing of cheomtherapy, verified against diagnosis date and end of pregnancy date. | 1^st^ trimester (<12 weeks)/  2^nd^ trimester (13-27 weeks)/  3^rd^ trimester (28-40+ weeks) |

**Supplementary Table 2**

NHS Trusts contributing patients:

**England**

Buckinghamshire Healthcare NHS Trust

Cambridge University Hospitals NHS Foundation Trust

Oxford University Hospitals NHS Foundation Trust

Royal Surrey County Hospital NHS Trust

Royal United Hospitals Bath NHS Foundation Trust

Sheffield Teaching Hospitals NHS Foundation Trust

Birmingham University Hospitals NHS Trust.

The Christie NHS Foundation Trust, Manchester

The Newcastle Upon Tyne Hospitals NHS Foundation Trust

University Hospitals Bristol and Weston NHS Foundation Trust

**Scotland**

NHS Greater Glasgow and Clyde

NHS Tayside

NHS Lothian

**Wales**

Swansea Bay NHS Trust

**Supplementary Figure 1**. Cases by year of diagnosis
